# Factorial Design Approach in Proportioning Prestressed Self-Compacting Concrete

**DOI:** 10.3390/ma8031089

**Published:** 2015-03-13

**Authors:** Wu-Jian Long, Kamal Henri Khayat, Guillaume Lemieux, Feng Xing, Wei-Lun Wang

**Affiliations:** 1 Guangdong Province Key Laboratory of Durability for Marine Civil Engineering, College of Civil Engineering, Shenzhen University, Shenzhen 518060, China; E-Mails: longwj@szu.edu.cn (W.-J.L.); xingf@szu.edu.cn (F.X.); 2 Faculty of Civil, Architectural and Environmental Engineering, Missouri University of Science and Technology, Rolla, MO 65409, USA; E-Mail: khayatk@mst.edu; 3 Cement Association of Canada, Montreal, QC J7H 1S7, Canada; E-Mail: glemieux@cement.ca

**Keywords:** self-compacting concrete, factorial design, statistical model, model region, mechanical properties, visco-elastic properties

## Abstract

In order to model the effect of mixture parameters and material properties on the hardened properties of, prestressed self-compacting concrete (SCC), and also to investigate the extensions of the statistical models, a factorial design was employed to identify the relative significance of these primary parameters and their interactions in terms of the mechanical and visco-elastic properties of SCC. In addition to the 16 fractional factorial mixtures evaluated in the modeled region of −1 to +1, eight axial mixtures were prepared at extreme values of −2 and +2 with the other variables maintained at the central points. Four replicate central mixtures were also evaluated. The effects of five mixture parameters, including binder type, binder content, dosage of viscosity-modifying admixture (VMA), water-cementitious material ratio (w/cm), and sand-to-total aggregate ratio (S/A) on compressive strength, modulus of elasticity, as well as autogenous and drying shrinkage are discussed. The applications of the models to better understand trade-offs between mixture parameters and carry out comparisons among various responses are also highlighted. A logical design approach would be to use the existing model to predict the optimal design, and then run selected tests to quantify the influence of the new binder on the model.

## 1. Introduction

Self-compacting concrete (SCC) is a highly workable concrete that can flow through densely reinforced or geometrically complex structural elements under its own weight and adequately fill voids without segregation or apparent bleeding, requiring no vibration for its consolidation [1,2,3]. Successful use of SCC in precast, prestressed and cast-in-place applications worldwide may help in the construction of longer-lived and more cost-effective structures [4,5,6,7,8,9,10].

Although the mix design of concrete is critical to its workability and performance, adequate selection of material constituents is a key factor in the optimization process of a concrete mixture that can achieve adequate performance and service life [11,12]. Material characteristics and mix design of SCC have a marked effect on all aspects of SCC production and placement, and on the fresh and hardened properties of the concrete [13,14]. The proportioning of SCC often involves the adjustment of several mixture parameters to achieve a compromise between the properties in its fresh and hardened states [15]. While certain design methods and mathematical approaches for SCC have been published [16,17,18,19,20,21,22,23,24,25,26], a lack of adequate research studies warrants investigation of SCC designated for prestressed applications.

In order to better understand the influence of key mix design parameters and material constituents on the behaviour of SCC, and also to investigate the extensions of the statistical models, a fractional factorial design was used to identify the relative significance of these primary parameters and their interactions with the mechanical and visco-elastic properties of prestressed SCC. A total of 28 SCC mixture combinations were used in the experimental design. It is important to note that the research work presented herein is an extension of the previous experimental plan presented by the authors [15]. The applications of the models to better understand trade-offs between mixture parameters and carry out comparisons among various responses are also highlighted. Better understanding of these parameters and their effects on the performance of SCC designated for prestressed applications and knowledge of trade-offs among the various mixture parameters on various properties of such concrete could simplify the test protocol needed to optimize SCC given a certain set of performance requirements, and therefore it is essential for successful development of prestressed SCC.

## 2. Experimental Program

### 2.1. Fractional Factorial Design

As shown in Table 1, a total of 28 SCC mixture combinations were used in the experimental design. A 2^5-1^ fractional factorial design was used to evaluate the influence of mixture proportioning and constituent material characteristics on the hardened properties of SCC. By definition, 2 is the number of levels of each factor investigated, 5 is the number of factors investigated, and 1 is the size of the fraction of the full factorial used. The first 16 mixtures for the fractional factorial plan were set at coded values of −1 and +1. The 2^5-1^ fractional factorial design was then expanded to include eight additional mixtures (axial mixtures) where each variable was adjusted separately at the extreme α value of −2 and +2 with the other variables maintained at the central points (0). This is done to extend the model’s values for the five principle variables and consider the quadratic effects for each variable. Four replicate central mixtures were also prepared to estimate the degree of the experimental error for the modeled responses.

**Table 1:** Details of experimental program.

	Type	Mix No.	Coded Values					Absolute Values				
			Binder	w/cm	VMA *	Binder Type	S/A **	Binder (kg/m^3^)	w/cm	VMA (mL/100 kg CM)	Binder Type	S/A (%)
Factorial mixtures	Fractional factorial points	1	−1	−1	−1	−1	1	440	0.34	0	MS	0.54
		2	−1	−1	−1	1	−1	440	0.34	0	HE ***	0.46
		3	−1	−1	1	−1	−1	440	0.34	100	MS	0.46
		4	−1	−1	1	1	1	440	0.34	100	HE	0.54
		5	−1	1	−1	−1	−1	440	0.40	0	MS	0.46
		6	−1	1	−1	1	1	440	0.40	0	HE	0.54
		7	−1	1	1	−1	1	440	0.40	100	MS	0.54
		8	−1	1	1	1	−1	440	0.40	100	HE	0.46
		9	1	−1	−1	−1	−1	500	0.34	0	MS	0.46
		10	1	−1	−1	1	1	500	0.34	0	HE	0.54
		11	1	−1	1	−1	1	500	0.34	100	MS	0.54
		12	1	−1	1	1	−1	500	0.34	100	HE	0.46
		13	1	1	−1	−1	1	500	0.40	0	MS	0.54
		14	1	1	−1	1	−1	500	0.40	0	HE	0.46
		15	1	1	1	−1	−1	500	0.40	100	MS	0.46
		16	1	1	1	1	1	500	0.40	100	HE	0.54
	Axial points	A1	−2	0	0	0	0	410	0.37	50	MS-HE	0.50
		A2	2	0	0	0	0	530	0.37	50	MS-HE	0.50
		A3	0	−2	0	0	0	470	0.31	50	MS-HE	0.50
		A4	0	2	0	0	0	470	0.43	50	MS-HE	0.50
		A5	0	0	−2	0	0	470	0.37	0	MS-HE	0.50
		A6	0	0	2	0	0	470	0.37	150	MS-HE	0.50
		A7	0	0	0	0	−2	470	0.37	50	MS-HE	0.42
		A8	0	0	0	0	2	470	0.37	50	MS-HE	0.58
	Central points	C1	0	0	0	0	0	470	0.37	50	MS-HE	0.50
		C2	0	0	0	0	0	470	0.37	50	MS-HE	0.50
		C3	0	0	0	0	0	470	0.37	50	MS-HE	0.50
		C4	0	0	0	0	0	470	0.37	50	MS-HE	0.50

* Thickening-type viscosity-modifying admixture (VMA); ** Crushed aggregate with MSA (maximum size of aggregate) of 12.5 mm and natural sand; *** Type HE cement + 20% Class F fly ash.

**Figure 1:**
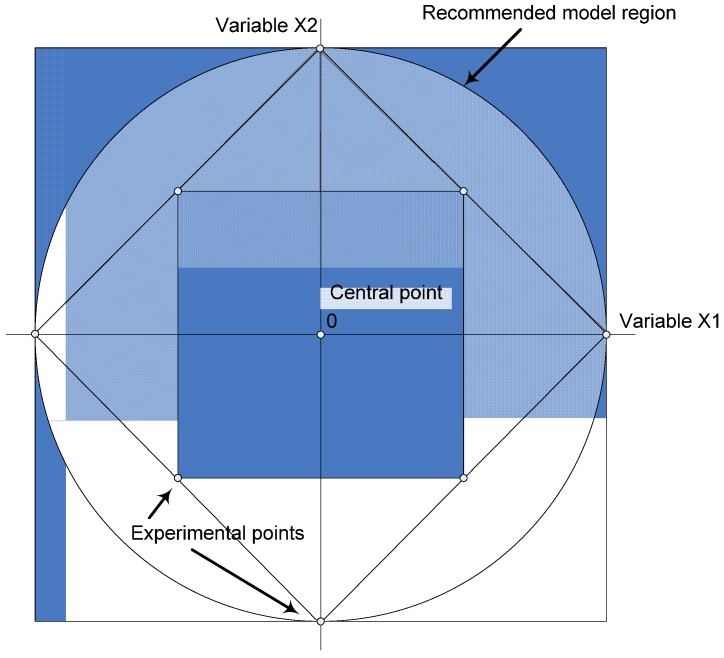
Presentation of modeled region.

The five modeled mixture parameters included the binder content (BC), binder type (BT), water-to-cementitious materials ratio (w/cm), dosage of thickening-type viscosity modifying admixture (VMA), and volume of the sand-to-total aggregate ratio (S/A). The experimental modeled region is illustrated in Figure 1. The modeled responses within the model region of −2 to +2 include compressive strength, modulus of elasticity, as well as autogenous and drying shrinkage. It is important to note that the objective of enlarging the statistical models to −2 and +2 is to attempt to improve the quality of the models and consider any quadratic effects of certain variables.

The coded and absolute values used for the factorial design are presented in Table 1. The coded values are calculated as the difference between the absolute values and values corresponding to the central points, divided by the spread between the absolute values corresponding to 0 and 1, as shown below:

Coded BC = (absolute BC–470)/30
Coded w/cm = (absolute w/cm–0.37)/0.03
Coded VMA = (absolute VMA–50)/50
Coded S/A = (absolute S/A–0.50)/0.04

The statistical models are valid for mixtures between −2 and +2 consisting of mixtures with a w/cm of 0.31 to 0.43, binder content from 410 to 530 kg/m^3^, VMA of 0 to 150 mL/100 kg CM (cementitious material), and S/A between 0.42 and 0.58, by volume. The ranges of these variables were selected to cover a wide scope of mixture ingredients. The choices of the w/cm and binder type were based on the results obtained in the previous parametric study [11]. A low w/cm was included to secure superior mechanical performance and the higher w/cm for better workability. Type HE (high early strength) binder with 20% Class F fly ash replacement was chosen given its better overall performance in terms of workability and compressive strength development compared to SCC made with Type HE binder with 30% slag [11]. The coarse aggregate employed for the experimental design was crushed aggregate with a maximum size of coarse aggregate (MSA) of 12.5 mm. This aggregate was shown to offer better performance in terms of workability and strength development than gravel of similar MSA or crushed aggregate with MSA 9.5 or 19 mm [11].

Factorial designs are made to test two or more factors at the same time. This is done by comparing the results obtained with different levels of each factor. The major benefit of using this approach is to find the combined effects of various parameters [27].

### 2.2. Materials Characteristics

Two types of Portland cement, Type MS (Moderate Sulfate) and Type HE, and a Class F fly ash were used in this investigation. The specific gravities of the MS cement, HE cement, and fly ash are 3.14, 3.15, and 2.53, respectively, and their Blaine fineness values are 390, 530, and 410 m^2^/kg, respectively. The SCC mixtures were evaluated using Type MS cement without any fly ash, as well as Type HE cement with 20% Class F fly ash replacement, by mass.

All concrete mixtures were prepared with crushed aggregate with a 12.5 mm MSA. Natural siliceous sand with a specific gravity of 2.66 conforming to AASHTO T 27 specifications was used [28]. The particle-size distribution of the sand and coarse aggregate are within the AASHTO recommended limits.

A polycarboxylate-based HRWRA (high-range water-reducing admixture) complying with ASTM C494C/C494M (Type F) and an organic, thickening-type VMA were used in the SCC mixtures [29]. The specific gravities of these admixtures are 1.047 and 1.0, respectively, and their solid contents are 20.3% and 6%, respectively.

### 2.3. Mixing Sequence

**Figure 2:**
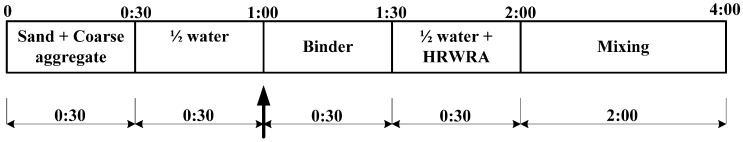
Standard mixing sequence.

The SCC mixtures were prepared using a drum mixer. The mixing sequence consisted of wetting the sand and coarse aggregate with half of the mixing water, followed by the addition of the binder. The initial wetting of the aggregate was carried out to ensure that the aggregate could later be coated by a layer of cement paste that would enhance the quality of the interface between the aggregate and hydrated cement paste. The HRWRA and VMA diluted with the remaining mixing water were then introduced over 30 s, and the concrete was mixed for 2.5 min, as shown in Figure 2. The concrete remained at rest in the mixer for 2 min for fluidity adjustment and to enable any large air bubbles trapped during mixing to rise to the surface.

### 2.4. Test Methods

Concrete cylinders measuring 100 × 200 mm and 150 × 300 mm were sampled 10 min after the end of mixing according to ASTM C192-14/C192M–14, to evaluate compressive strength, modulus of elasticity, and autogenous and drying shrinkage under three different curing conditions summarized in Table 2 [30,31,32,33]. The samples used to determine compressive strength and elastic modulus were air-cured and steam-cured for the first 18 h, and also moist-cured for 7, 28, and 56 days.

**Table 2:** Curing conditions.

Curing Method	Stage	Detail
Steam-curing	I	Ambient temperature for 2 h after water-cement contact
	II	Temperature raised for 2 h
	III	Concrete temperature maintained for 10 h
	IV	Temperature decreases over 2 h to ambient temperature
	V	Air-curing until age of testing at 18 h
Moist-curing	I	18 h in molds with wet burlap at 23 ± 2 °C
	II	Moist-cured at 23 ± 2 °C until testing age
Air-curing	I	18 h in molds with wet burlap at 23 ± 2 °C
	II	Air-dried at 23 ± 2 °C until testing age

**Figure 3:**
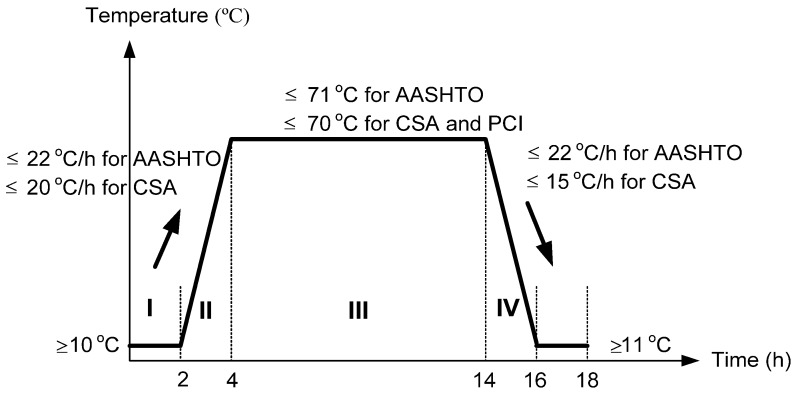
Steam curing regime specified by AASHTO and CSA.

All sampled specimens were cast without any mechanical consolidation. Some of the samples were covered and remained in the laboratory at 23 ± 2 °C to air cure until the time of testing, while others were steam-cured according to the regime described in Figure 3. Based on the AASHTO (American Association of State Highway and Transportation Officials), CSA (Canadian Standards Association), and PCI (Precast/Prestressed Concrete Institute) specifications, the maximum curing temperature in the concrete should not exceed 70 °C to prevent the occurrence of delayed ettringite formation [34,35,36]. The standards also stipulate that the increase in temperature-time ratio should not exceed 22 and 20 °C/h (depending on the standard), and the decrease in temperature-time ratio should be lower than 22 and 15 °C/h for the AASHTO and CSA, respectively.

All of the investigated mixtures considered in the experimental design had an initial slump flow of 680 ± 20 mm that was obtained by adjusting the dosage rate of the high-range water reducing admixture (HRWRA). The targeted release compressive strength after 18 h of steam curing and 56-day of moist curing were 35 MPa and 55 to 70 MPa, respectively. The compressive strength was determined on 100 × 200 mm cylinders. For 56-day compressive strength, the specimens were stored at 100% relative humidity and 23 ± 2 °C until the time of testing. In addition to compressive strength, samples were subjected to steam curing to determine the 18-h modulus of elasticity and to initiate shrinkage testing at the prestress release time. The compressive strengths and the modulus of elasticity of the tested SCC at various ages are presented in Table 3 and Table 4, respectively.

Autogenous shrinkage was measured on prisms measuring 75 × 75 × 285 mm. The prisms were sealed immediately after removal from the molds at 18 h of age and kept at 23 ± 2 °C until the end of testing. Autogenous shrinkage was monitored using embedded vibrating wire strain gauges until stabilization.

Six 150 × 300 mm cylindrical test specimens were cast to monitor drying shrinkage. The specimens were steam cured until the age of 16 h and were then demolded. The ends of the cylinders were ground, and external studs were installed for deformation measurements. A digital-type extensometer was used to determine drying shrinkage. Drying shrinkage testing started at the age of 18 h. Shrinkage specimens were kept in a temperature-controlled room at 23 ± 2 °C and 50% ± 4% relative humidity. Drying shrinkage deformations were monitored for 11 months. The detailed autogenous and drying shrinkage values at various ages are given in Table 5.

**Table 3:** Compressive strength of the tested SCC (MPa).

Mix ID		18 h	7 Days	28 Days	56 Days
		Steam-Cured	Moist-Cured		
Fractional factorial points	1	36.8	54.2	58.8	60.7
	2	33.0	48.0	59.2	62.5
	3	38.0	54.6	60.4	69.6
	4	31.5	51.6	60.2	65.3
	5	31.9	44.6	51.2	53.2
	6	33.3	46.2	54.2	61.0
	7	28.1	41.5	49.8	55.7
	8	31.8	49.6	56.3	66.9
	9	35.1	51.7	62.2	68.2
	10	33.8	55.7	64.8	78.0
	11	36.6	57.5	64.5	69.1
	12	33.5	57.7	70.2	75.4
	13	31.8	44.9	51.2	58.2
	14	30.9	40.4	55.2	60.9
	15	30.1	37.6	47.5	55.4
	16	28.1	39.0	47.6	53.2
Axial points	A1	30.6	55.3	66.6	70.7
	A2	37.6	50.3	59.4	65.2
	A3	8.5	65.9	75.8	81.5
	A4	29.4	39.9	48.7	53.4
	A5	30.4	53.7	63.7	69.0
	A6	33.0	53.9	61.1	68.8
	A7	36.0	52.9	63.3	66.1
	A8	35.6	49.5	60.1	63.7
Central points	C1	36.0	52.7	65.0	71.6
	C2	36.0	52.0	64.2	71.7
	C3	36.5	54.3	63.8	69.2
	C4	35.1	51.1	62.1	69.1

**Table 4:** Modulus of elasticity and HRWRA demand of the tested SCC.

Mix ID		18 h	28 Days	56 Days	HRWRA Demand
		Steam-Cured	Moist-Cured		L/100 kg CM
		GPa	GPa	GPa	
Fractional factorial points	1	33.0	37.0	39.5	2.23
	2	31.0	39.0	41.5	2.73
	3	34.0	41.0	42.5	2.39
	4	29.0	37.5	38.0	2.95
	5	30.0	34.5	36.5	0.77
	6	29.5	35.5	38.0	1.20
	7	30.5	34.0	34.0	0.86
	8	31.0	37.5	39.0	1.41
	9	32.5	39.5	41.0	1.80
	10	31.5	39.0	40.5	2.00
	11	33.0	38.0	39.0	2.00
	12	34.5	41.0	41.5	2.00
	13	28.5	34.0	35.0	0.50
	14	27.5	37.0	38.5	1.02
	15	28.0	34.0	35.0	0.70
	16	24.0	32.5	33.5	1.00
Axial points	A1	30.5	38.0	40.0	1.13
	A2	28.0	35.0	35.5	2.47
	A3	18.5	40.0	41.0	3.95
	A4	27.0	32.5	34.5	0.70
	A5	28.0	37.5	37.5	1.67
	A6	30.5	35.5	38.5	1.73
	A7	29.5	36.0	38.5	1.43
	A8	30.0	35.5	37.0	1.53
Central points	C1	28.5	37.0	38.5	1.73
	C2	30.0	37.5	39.5	1.73
	C3	29.0	38.5	39.5	1.73
	C4	30.0	38.5	38.5	1.73

## 3. Results and Discussion

### 3.1. Derived Statistical Models for Mechanical Properties

Statistical models were established by multi-regression analyses. Mean values and standard deviations for each of the responses and calculated relative errors corresponding to 90% confidence limits are summarized in Table 6.

The coefficient and probality (Prob.) > |**t**| values of the derived models for compressive strength and modulus of elasticity (MOE) are presented in Table 7. The estimate for each factor refers to the contribution of that factor to the modeled response. Probability values less than 0.1 were considered significant evidence that the factor has a significant influence on the modeled response. Student tests were run to evaluate the significance of the model factors and their second-order interactions on a given response. For each modeled response, the single-operator relative error corresponding to a 90% confidence limit was used to perform the significance evaluation. Single-operator relative errors were determined using a mixture corresponding to the central point of the experimental design.

**Table 5:** Autogenous and drying shrinkage values at various ages (μstrain).

Mixture ID		Autogenous Shrinkage		Drying Shrinkage			
		7 Days	28 Days	7 Days	28 Days	112 Days	250 Days
Fractional factorial points	1	75	105	270	465	720	830
	2	250	265	180	310	435	535
	3	85	115	160	270	450	565
	4	205	260	240	365	615	735
	5	35	90	80	130	330	495
	6	95	190	130	230	475	545
	7	50	100	80	195	440	585
	8	130	245	160	270	570	670
	9	130	155	290	550	820	975
	10	245	315	315	540	765	930
	11	150	230	185	255	530	680
	12	190	305	235	330	540	635
	13	70	110	140	225	540	690
	14	70	140	150	280	555	640
	15	65	100	100	190	405	545
	16	75	170	150	380	620	720
Axial points	A1	135	220	140	295	420	520
	A2	125	230	175	390	550	660
	A3	180	225	215	355	450	515
	A4	70	140	165	375	555	675
	A5	145	220	150	275	430	565
	A6	120	165	160	330	460	575
	A7	125	200	215	350	470	565
	A8	115	190	190	350	490	590
Central points	C1	120	200	190	330	500	615
	C2	115	200	230	340	515	620
	C3	110	200	190	370	520	615
	C4	120	200	180	345	495	620

**Table 6:** Mean values and relative errors of central points (90% confidence limit).

Property	Mean	Standard Deviation	Relative Error * in 90% Confidence Limit, (%)
18 h compressive strength, MPa	35.9	0.583	1.9
28 days compressive strength, MPa	63.8	1.223	2.3
56 days compressive strength, MPa	70.4	1.445	2.4
18 h modulus of elasticity, GPa	29.4	0.750	3.0
28 days modulus of elasticity, GPa	37.9	0.750	2.3
56 days modulus of elasticity, GPa	39.0	0.550	1.7

* Relative error = $2.35\frac{\text{σ}}{\bar{x}\sqrt{n}}\cdot 100\;(\%)$; where, 2.35 = coefficient representing the 90% confidence interval for the Student distribution for *n* = 4; σ = standard deviation; *n* = number of observations; $\bar{x}$ = mean value of observations.

**Table 7:** Parameter estimates of derived models for mechanical properties.

**Property**	**7-Day $f_{c}^{\prime }$, MPa *R^2^* = 0.76**		**28-Day $f_{c}^{\prime }$, MPa *R^2^* = 0.72**		**56-Day $f_{c}^{\prime }$, MPa *R^2^* = 0.79**	
**Model Type**	**Linear Model**		**Linear Model**		**Linear Model**	
**Parameters**	**Estimates**	**Prob. > |t|**	**Estimates**	**Prob. > |t|**	**Estimates**	**Prob. > |t|**
Intercept	+50.25	-	+59.62	-	+65.43	-
Binder content	NS *	NS	NS	NS	NS	NS
w/cm	−5.80	0.001	−5.90	0.001	−5.85	0.001
VMA	NS	NS	NS	NS	NS	NS
Binder type	NS	NS	+1.50	0.061	+2.52	0.006
S/A	NS	NS	NS	NS	NS	NS
BC·w/cm	−2.13	0.017	−2.08	0.035	−2.60	0.018
BC·VMA	NS	NS	NS	NS	−2.02	0.062
w/cm·BT	NS	NS	NS	NS	NS	NS
**Property**	**18-h MOE, GPa (Steam-Cured) *R^2^* = 0.73**		**28-Day MOE, GPa *R^2^* = 0.75**		**56-Day MOE, GPa *R^2^* = 0.85**	
**Model Type**	**Linear Model**		**Linear Model**		**Linear Model**	
**Parameters**	**Estimates**	**Prob. > |t|**	**Estimates**	**Prob. > |t|**	**Estimates**	**Prob. > |t|**
Intercept	+29.92	-	+36.83	-	+38.24	-
Binder content	−0.56	0.040	−0.29	0.100	−0.55	0.014
w/cm	−1.48	0.001	−2.00	0.001	−1.97	0.001
Binder type	−0.40	0.100	+0.50	0.051	+0.57	0.012
S/A	NS	NS	−0.71	0.008	−0.89	0.001
BC·w/cm	−1.09	0.002	NS	NS	NS	NS
w/cm·BT	NS	NS	NS	NS	+0.54	0.045
VMA·S/A	−0.78	0.020	NS	NS	−0.52	0.055
BT·S/A	−0.66	0.050	NS	NS	NS	NS

* NS: Not significant.

The derived models are summarized in Table 8 with the mixture variables expressed as coded values. The models are expressed as the factors with the highest influence on the modeled responses list in descending order. A negative estimate signifies that an increase in the modeled parameters results in a reduction in the measured response. For example, in the case of 7-day compressive strength model, an increase in w/cm is expected to decrease the 7-day compressive strength.

**Table 8:** Derived statistical models for mechanical properties.

Property	Age	Derived Equations	*R^2^*
Compressive strength, MPa	7 days	+50.25 − 5.80 w/cm − 2.13 (BC·w/cm)	0.76
	28 days	+59.62 − 5.90 w/cm + 1.50 BT − 2.08 (BC·w/cm)	0.72
	56 days	+65.43 − 5.85 w/cm + 2.52 BT − 2.60 (BC·w/cm) − 2.02 (BC·VMA)	0.79
Modulus of elasticity, GPa	18 h	+29.92 − 1.48 w/cm − 0.56 BC − 0.40 BT − 1.09 (BC·w/cm) − 0.78 (VMA·S/A) − 0.66 (BT·S/A)	0.73
	28 day	+36.83 − 2.00 w/cm − 0.71 S/A + 0.50 BT − 0.29 BC	0.75
	56 day	+38.24 − 1.97 w/cm − 0.89 S/A + 0.57 BT − 0.55 BC + 0.54 (w/cm·BT) – 0.52 (VMA·S/A)	0.85

The w/cm had the greatest influence on investigated mechanical properties. The binder type (BT) had considerable effect on compressive strength and MOE. The long-term MOE response (28 days and 56 days) appeared to be significantly affected by S/A. In most cases, the use of VMA did not have significant effect on mechanical properties.

Based on the derived models for mechanical properties for the modeled region between −2 and +2, the main findings can be summarized as follows:

- Compressive strength and MOE are shown, as expected, to increase as the decrease in w/cm; furthermore, the increase in binder content leads to a decrease in MOE.
- The increase in S/A has negative effect on MOE at 28 and 56 days (moist curing).
- Type HE cement and 20% of Class F fly ash exhibit higher compressive strength and MOE at 28 and 56 days but lower mechanical properties at 18 h compared to Type MS cement.

### 3.2. Evaluation of Statistical Models for Mechanical Properties

**Figure 4:**
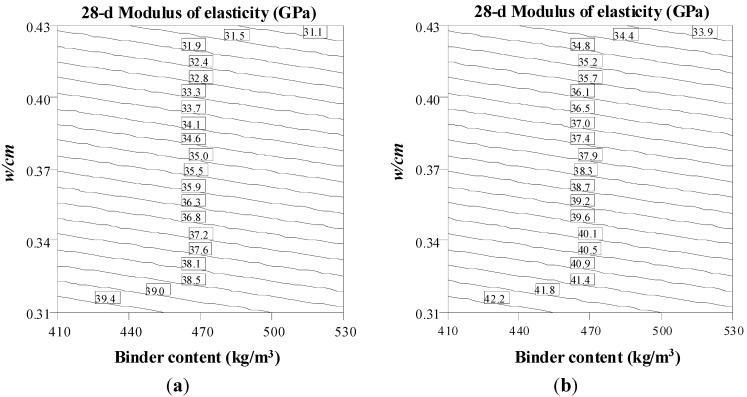

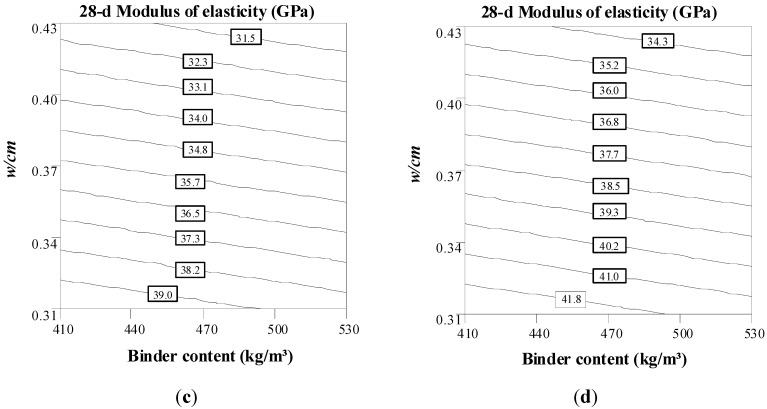
Binder content − w/cm contour diagrams of 28-day modulus of elasticity (VMA = 50 mL/100 kg CM). (**a**) S/A = 0.58, Type MS; (**b**) S/A = 0.42, Type MS; (**c**) S/A = 0.58, Type HE + 20% FA; (**d**) S/A = 0.42, Type HE + 20% FA.

The contour diagrams of the 28-day modulus of elasticity in Figure 4 illustrate the trade-offs between binder content and w/cm for mixtures made with Type MS and Type HE with 20% fly ash binder and 0.42 and 0.58 S/A, corresponding to coded values of −2 and +2. The VMA value is set to the central points. As indicated in Figure 4, for the same w/cm and binder content, the S/A and type of binder have no significant effect on MOE at 28 days. As expected, the increase in both binder content and w/cm decreases the MOE. For example, for SCC made with 500 kg/m^3^ binder content, Type MS cement, and 0.58 S/A, the increase in w/cm from 0.34 to 0.40 leads to the decrease of 28-day modulus of elasticity from 37 to 32.5 GPa, as shown in Figure 4a.

### 3.3. Derived Statistical Models for Autogenous and Drying Shrinkage

Mean values and standard deviations for the autogenous and drying shrinkage responses and the calculated relative errors corresponding to 90% confidence limits are summarized in Table 9. The coefficients and Prob. > |**t**| values of the derived models for autogenous and drying shrinkage are presented in Table 10.

**Table 9:** Mean values and relative errors of central points (90% confidence limit).

Property	Mean	Standard Deviation	Relative Error * in 90% Confidence Limit, (%)
7 days autogenous shrinkage, μstrain	115	4.8	4.8
28 days autogenous shrinkage, μstrain	200	2.5	1.5
56 days autogenous shrinkage, μstrain	230	2.9	1.5
28 days drying shrinkage, μstrain	345	17.0	5.8
112 days drying shrinkage, μstrain	505	11.9	2.8
250 days drying shrinkage, μstrain	600	5.8	1.1

* Relative error = $2.35\frac{\text{σ}}{\bar{x}\sqrt{n}}\cdot 100\;(\%)$; where, 2.35 = coefficient representing the 90% confidence interval for the Student’s distribution for *n* = 4; σ = standard deviation; *n* = number of observations; $\bar{x}$ = mean value of observations.

**Table 10:** Parameter estimates of derived models for autogenous and drying shrinkage.

**Property**	**Autogenous Shrinkage at 7 Days (μstrain) *R^2^* = 0.92**		**Autogenous Shrinkage at 56 Days (μstrain) *R^2^* = 0.82**		**Drying Shrinkage at 7 Days (μstrain) *R^2^* = 0.70**	
**Model Type**	**Linear Model**		**Linear Model**		**Linear Model**	
**Parameters**	**Estimates**	**Prob. > |t|**	**Estimates**	**Prob. > |t|**	**Estimates**	**Prob. > |t|**
Intercept	+129.87	-	+217.0	-	+173.23	-
Binder content	NS *	NS	+8.33	0.100	+16.21	0.068
w/cm	−37.42	0.001	−37.5	0.001	−43.46	0.001
Binder type	+30.75	0.001	+54.75	0.001	NS	NS
BC·w/cm	−21.63	0.001	−18.75	0.033	NS	NS
BC·BT	−15.88	0.001	NS	NS	NS	NS
BC·S/A	+9.88	0.001	+17.75	0.043	NS	NS
BC·VMA	NS	NS	NS	NS	−18.56	0.087
w/cm·VMA	NS	NS	NS	NS	+20.69	0.058
w/cm·BT	−20.13	0.001	NS	NS	NS	NS
**Property**	**Drying shrinkage at 28 Days (μstrain) *R^2^* = 0.72**		**Drying shrinkage at 112 Days (μstrain) *R^2^* = 0.82**		**Drying shrinkage at 250 Days (μstrain) *R^2^* = 0.73**	
**Model Type**	**Linear Model**		**Linear Model**		**Linear Model**	
**Parameters**	**Estimates**	**Prob. > |t|**	**Estimates**	**Prob. > |t|**	**Estimates**	**Prob. > |t|**
Intercept	+323.23	-	+517.77	-	+620.00	-
Binder content	+31.54	0.024	+41.33	0.009	+47.50	0.006
w/cm	−45.71	0.002	−36.25	0.019	−39.17	0.021
S/A	NS	NS	+26.58	0.078	+29.75	0.072
BC·VMA	−28.19	0.091	−44.38	0.019	−51.00	0.015
w/cm·VMA	+48.44	0.006	+45.13	0.018	+50.75	0.015
w/cm·BT	+28.81	0.084	+41.88	0.026	NS	NS
VMA·BT	+30.81	0.066	+42.63	0.024	+47.63	0.022

* NS: Not significant.

Based on the statistical models established in this investigation, the w/cm had the most significant influence on autogenous and drying shrinkage at various ages. The type of binder had considerable effect on autogenous shrinkage. Drying shrinkage varied mainly with the binder content. The S/A value had considerable effect on drying shrinkage at 112 and 250 days. In most cases, VMA had a minor effect on the measured responses. The derived models are summarized in Table 11. The main findings from the statistical models within the −2 to +2 region are given as follows:

- Autogenous shrinkage of SCC decreases as w/cm increases.
- Increase in binder content leads to an increase in drying shrinkage.
- For a given binder content, drying shrinkage of concrete does not increase with w/cm*.* This is because the drying shrinkage measurement also includes autogenous shrinkage, which obviously decreases with the increase in w/cm.
- SCC made with Type HE cement and 20% Class F fly ash develops higher autogenous shrinkage compared to concrete prepared with Type MS cement.
- Binder type and thickening-type VMA do not have a significant effect on drying shrinkage.

**Table 11:** Derived statistical models for autogenous and drying shrinkage.

Property	Age	Derived Equations	*R^2^*
Autogenous shrinkage, μstrain	7 days	+129.9 − 37.4 w/cm + 30.8 BT − 21.6 (BC·w/cm) − 20.1 (w/cm·BT) − 15.9 (BC·BT) + 9.9 (BC·S/A)	0.92
	56 days	+217.0 + 54.8BT − 37.5 w/cm + 8.3 BC − 18.8 (BC·w/cm) + 17.8 (BC·S/A)	0.82
Drying shrinkage, μstrain	7 days	+173.2 − 43.5 w/cm +16.2 BC − 18.6 (BC·VMA) + 20.7 (w/cm·VMA)	0.70
	28 days	+323.2 − 45.7 w/cm + 31.5 BC + 48.4 (w/cm·VMA) + 28.8 (w/cm·BT) − 28.2 (BC·VMA) + 30.8 (VMA· BT)	0.72
	112 days	+517.8 + 41.3 BC − 36.3 w/cm + 26.6 S/A + 45.1 (w/cm·VMA) − 44.4 (BC·VMA) + 42.6 (VMA·BT) + 41.9 (w/cm·BT)	0.82
	250 days	+620.0 + 47.5 BC − 39.2 w/cm + 29.8S/A − 51.0 (BC·VMA) + 50.8 (w/cm·VMA) + 47.6 (VMA·BT)	0.73

### 3.4. Evaluation of Statistical Models for Visco-Elastic Properties

**Figure 5:**
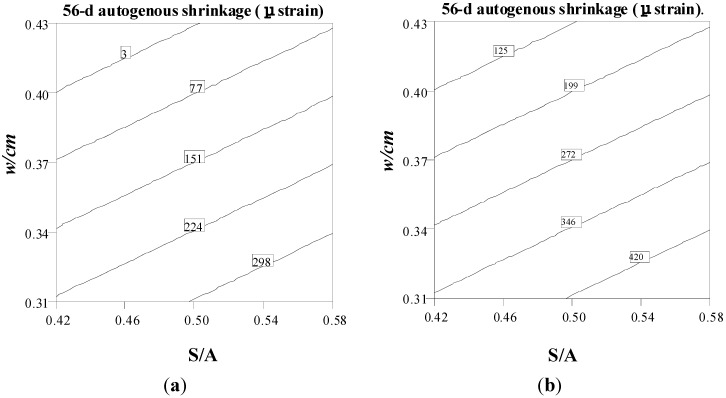
S/A–w/cm contour diagrams of 56-day autogenous shrinkage (Binder content = 530 kg/m^3^, VMA = 100 mL/100 kg CM). (**a**) Type MS; (**b**) Type HE + 20% FA.

Autogenous shrinkage contour diagrams at 56 days are presented in Figure 5 to show trade-offs between w/cm and S/A for mixtures made with Type MS cement and Type HE cement and 20% fly ash. The binder content and VMA content are set at 530 kg/m^3^ and 100 mL/100kg CM, respectively. As expected, for a constant S/A value, an increase in w/cm decreases autogenous shrinkage. Furthermore, for SCC made with the same S/A and w/cm, the use of Type HE cement and 20% fly ash results in higher autogenous shrinkage than mixtures prepared with Type MS cement.

**Figure 6:**
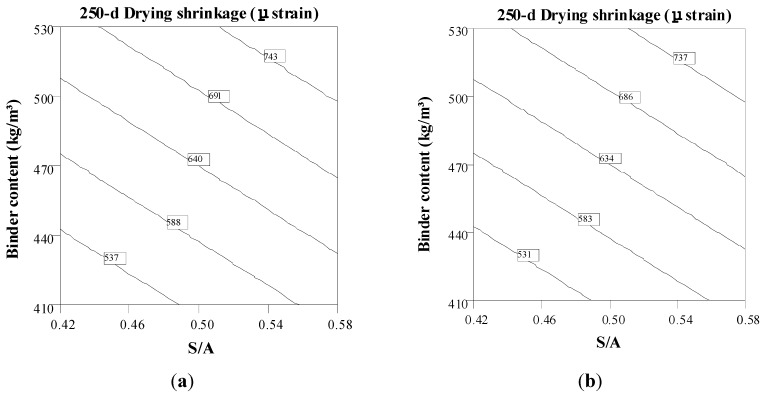
Binder content–S/A contour diagrams of 250-day drying shrinkage (w/cm = 0.37, VMA = 50 mL/100 kg CM). (**a**) Type MS; (**b**) Type HE + 20%FA.

The drying shrinkage contour diagrams at 250 days in Figure 6 illustrate the trade-offs between binder content and S/A for mixtures made with Type MS and Type HE cement with 20% fly ash. The w/cm and VMA values are set to the central points for both mixtures. In general, the increase in binder content leads to an increase in drying shrinkage. As illustrated in Figure 6a,b, for a constant S/A of 0.50, an increase in binder content from 440 to 500 kg/m^3^ leads to an increase in the 250-day drying shrinkage values from 590 to 690 μstrain and from 580 to 680 μstrain, for mixtures made with Type MS and Type HE with 20% fly ash binder, respectively. Moreover, SCC made with higher S/A exhibits higher drying shrinkage after 250 days of drying. No significant differences were found for SCC made with the same binder content and S/A but different binder types.

### 3.5. Extension of the Statistical Models

In this investigation, the 2^5-1^ fractional factorial design was expanded to include eight additional mixtures where each variable was adjusted separately at the extreme α value of −2 and +2, with other variables maintained at the 0 central points. The variations of *R^2^* values within the −1 and +1 and −2 and +2 modeled regions for the mechanical and visco-elastic property responses are summarized in Table 12. The *R^2^* values within the −1 and +1 region are obtained from the previous investigations [15]. The correlation coefficient *R^2^* values are shown to decrease when the modeled region varies from the (−1, +1) range to the (−2, +2) range. This is because the error in predicting each response increases with the deviation from the center of modeled region. Deviation from central points has a negative influence on the degree of prediction of the various models.

**Table 12:** Variations of *R^2^* values with different modeled regions.

Modeled Region	Autogenous Shrinkage		Drying Shrinkage		Compressive Strength	MOE	
	7 Days	56 Days	28 Days	112 Days	56 Days	18 h	56 Days
−1, +1	0.96	0.93	0.78	0.96	0.87	0.89	0.87
−2, +2	0.92	0.82	0.72	0.82	0.79	0.73	0.85

The accuracy of predicted responses is affected by deviation from the set of materials used in establishing the models. However, the models can still be used for mixture optimizations and simulations despite changes in material characteristics, since such materials have limited effect on the prediction accuracy of the modeled responses. A logical design approach would be to use the existing model to predict the optimal design, and then run selected tests to quantify the influence of the new binder on the model. A limited number of mixtures can be prepared to adjust the existing model to take into consideration the influence of the newly considered material types on concrete properties relevant to the quality of SCC.

## 4. Conclusions

The statistical models established using a factorial design approach can be used to quantify the effects of mixture parameters and their coupled effects on the fresh, mechanical, and visco-elastic properties of SCC. In this investigation, a factorial design was adopted within the model regions of −2 and +2 to mathematically model the influence of five parameters on compressive strength, modulus of elasticity, autogenous shrinkage, and drying shrinkage of prestressed SCC. Based on the statistical models derived from the factorial design, the following conclusions can be drawn:

- In terms of mechanical properties, w/cm had the highest effect on compressive strength and modulus of elasticity; moreover, the content and type of binder had a considerable effect on mechanical properties. The modulus of elasticity was also affected by the S/A, and VMA content did not show a significant effect on mechanical properties.
- In terms of visco-elastic properties, w/cm was found to be the most significant factor, and the binder type also had a significant effect on autogenous shrinkage. Furthermore, drying shrinkage varied mainly with the binder content; in most cases, the VMA had a minor effect on autogenous and drying shrinkage.
- Based on the results from the derived models that were extended to the −2 to +2 region, no significant difference can be found between the statistical models (−1 to +1) and those extended to −2 to +2. In order to eliminate the outer regions approaching the edges of the modeled region and to minimize the prediction error, the model region can be limited to coded values of −1.5 to +1.5.
